# Microsporogenesis in the triploid hybrid ‘Beilinxiongzhu 1#’ and detection of primary trisomy in 2*x* × 3 × *Populus* hybrids

**DOI:** 10.1186/s12870-023-04189-9

**Published:** 2023-04-04

**Authors:** Yaru Sang, Bo Kong, Phuong Uyen Do, Lexun Ma, Jiahua Du, Liang Li, Xuetong Cheng, Yifan Zhao, Qing Zhou, Jian Wu, Lianjun Song, Pingdong Zhang

**Affiliations:** 1 College of Biological Sciences and Technology, Beijing Forestry University, Beijing, 100083 China; 2 National Engineering Research Center of Tree Breeding and Ecological Restoration, Beijing Forestry University, Beijing, 100083 China; 3 Key Laboratory of Genetics and Breeding in Forest Trees and Ornamental Plants, Ministry of Education, Beijing Forestry University, Beijing, 100083 China; 4 Forest Tree Species Breeding Base of Weixian Country, Hebei, 054700 China

**Keywords:** Hybrid triploid *Populus*, Microsporogenesis, Meiotic abnormality, SSR marker, Primary trisomy

## Abstract

**Background:**

Primary trisomy is a powerful genetic tool in plants. However, trisomy has not been detected in *Populus* as a model system for tree and woody perennial plant biology.

**Results:**

In the present study, a backcross between *Populus alba* × *Populus glandulosa* ‘YXY 7#’ (2n = 2*x* = 38) and the triploid hybrid ‘Beilinxiongzhu 1#’ (2n = 3*x* = 57) based on the observation of microsporogenesis and an evaluation of the variations in pollen was conducted to create primary trisomy. Many abnormalities, such as premature migration of chromosomes, lagging of chromosomes, chromosome bridges, asymmetric separation, micronuclei, and premature cytokinesis, have been detected during meiosis of the triploid hybrid clone ‘Beilinxiongzhu 1#’. However, these abnormal behaviors did not result in completely aborted pollen. The pollen diameter of the triploid hybrid clone ‘Beilinxiongzhu 1#’ is bimodally distributed, which was similar to the chromosomal number of the backcross progeny. A total of 393 progeny were generated. We provide a protocol for determining the number of chromosomes in aneuploid progeny, and 19 distinct simple sequence repeat (SSR) primer pairs covering the entire *Populus* genome were developed. Primary trisomy 11 and trisomy 17 were detected in the 2*x* × 3 *x* hybrid using the SSR molecular markers and counting of somatic chromosomes.

**Conclusions:**

Nineteen distinct SSR primer pairs for determining chromosomal number in aneuploid individuals were developed, and two *Populus* trisomies were detected from 2*x* × 3 *x* hybrids by SSR markers and somatic chromosome counting. Our findings provide a powerful genetic tool to reveal the function of genes in *Populus*.

## Background

Diploid with one extra chromosome in the genome is called trisomy and is a type of aneuploidy. Liu and Qin [1] divided plant trisomy into primary trisomy, secondary trisomy, tertiary trisomy, and terminal trisomy based on differences in the extra chromosome [2]. Among them, primary trisomy is the most widely used genetics tool. Target chromosomes can be replaced and separated by primary trisomy. Primary trisomy in plants has been used to locate genes and linkage groups to specific chromosomes to construct a physical map of genes [3, 4]. In addition, hybridizing plants with primary trisomy creates many genetic variations [5]. Therefore, primary trisomy genes are crucial genetic material, and more attention should be paid to establishing and identifying primary trisomy in plants.

Primary trisomy sets have been established for many plants. For example, primary trisomy in *Nicotiana sylvestris* Speg. & Comes [6] and *Datura stramonium* L. [7] were selected from natural mutations, although it is difficult for plants to spontaneously produce primary trisomy. Physical and chemical factors have been explored to induce primary trisomy. Hu et al. [8] treated *Gossypium hirsutum* L. seeds with γ-rays and detected five trisomies from self-crossing progeny. Chromosome non-or ahead-of-time-disjunction induced by irradiation may be why trisomic plants are produced. Another way to obtain primary trisomy is to screen the offspring of 3*x* × 2 *x* or 2*x* × 3 *x *hybrids, taking advantage of the irregular allocation of chromosomes during triploid meiosis. Primary trisomy in some essential crops, such as tomato [9], *Zea mays* [10], indica [11], sugar beets [12], barley [13], canola [14], and millet [15], were generated using this method. Additionally, autotetraploid microspore culture is another method to select primary trisomy quickly and efficiently. A set of primary trisomies has been obtained from isolated microspore-cultured plantlets of tetraploid Chinese cabbage [16]. Some previous studies [1, 17] reported that a significant number of primary trisomies can be obtained during anther culture of homologous tetraploid rice. Liu and Qin [1] detected 272 primary trisomies by culturing tetraploid rice anthers. Primary trisomy has also been obtained in diploids that have undergone asynapsis or desynapsis [6].

The methods used to identify primary trisomy in plants include morphological identification [7, 18], karyotyping of somatic mitosis [12], karyotyping of meiocytes during pachytene [19, 20], karyotyping of chromosome banding [21], and identification of a trisomic translocation line [8]. The first three methods have not been widely used because of the operation difficulties or errors.

*Populus* is a model system for tree and woody perennial plant biology. *Populus* trees are widely cultivated throughout the northern hemisphere as a source of fiber, biomass, and lumber [22]. However, to our knowledge, trisomy has not been detected in *Populus*. Some important poplar varieties, including ‘Beilinxiongzhu 1#’, ‘Beilinxiongzhu 2#’, and ‘Zhonglin 46’ were found to be triploids after the development of the *Populus* triploid breeding program in China [23, 24]. Therefore, it is possible to generate primary trisomies by crossing a triploid with a diploid in *Populus*.

In the present study, we obtained 393 backcross progeny by crossing the pollen of the triploid hybrid clone ‘Beilinxiongzhu 1#’ with the female gametes of *P. alba* × *P. glandulosa* ‘YXY 7#’. Then, a protocol for determining the number of chromosomes in aneuploid individuals is provided, and two real trisomies were detected using simple sequence repeat (SSR) markers and counting of the somatic chromosomes. Our findings provide new insight into *Populus* breeding with new genetic material.

## Results

### Meiotic analysis of the pollen mother cells (PMCs) in the triploid hybrid clone ‘Beilinxiongzhu 1#’

**Fig. 1:**
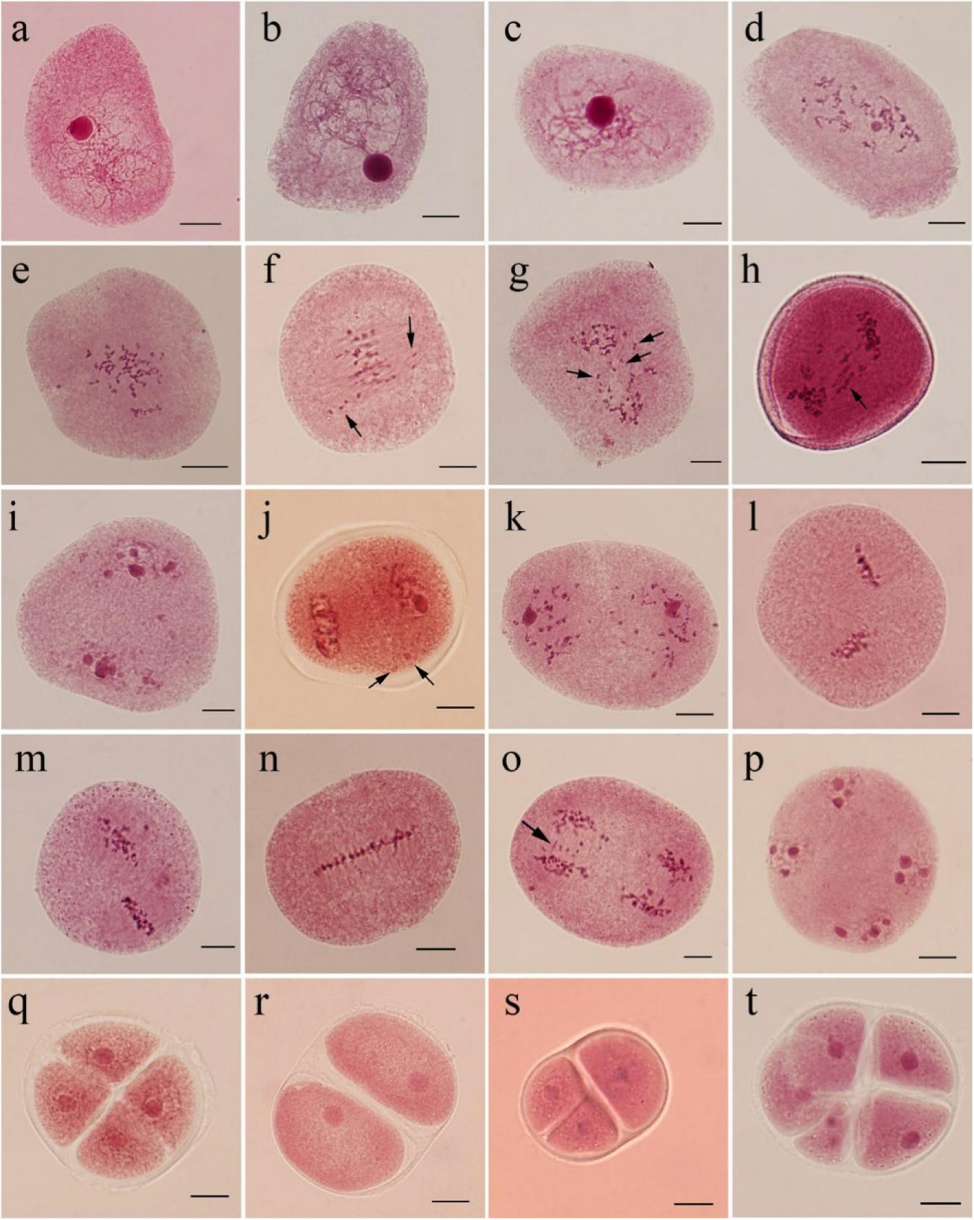
Meiosis of the PMCs in the triploid hybrid clone ‘Beilinxiongzhu 1#’. **a** Leptotene.** b** Zygotene. **c** Pachytene. **d** Diplotene. **e** Diakinesis. **f** Metaphase I arrow indicates a chromosome that migrated prematurely. **g** Lagging chromosomes at anaphase I (arrow). **h** Chromosome bridge at anaphase I (arrow). **i** Telophase I. **j** Micronuclei at telophase I (arrow). **k** Prophase II. **l** Metaphase II. **m** Parallel spindles at metaphase II. **n** Fused spindles at metaphase II. **o** Lagging chromosomes at anaphase II (arrow). **p** Telophase II. **q** Tetrad. **r** Dyad. **s** Triad. **t** Polyad. Bars, 10 μm

Meiosis of the PMCs in the triploid hybrid clone ‘Beilinxiongzhu 1#’ was a consecutive and asynchronous process (Table 1). Similar to other diploid *Populus* species, more than one meiotic stage was found in each male bud because of the asynchronous development of the PMCs in the different anthers. Abnormal phenomena often occur in the triploid hybrid poplar clone ‘Beilinxiongzhu 1#’ compared with diploid *Populus* species (Fig. 1).

**Table 1:** Meiosis of the PMCs in the triploid hybrid clone ‘Beilinxiongzhu 1#’

Meiotic stage of PMCs	Hours after being cultured												
	**142**	**168**	**180**	**188**	**192**	**196**	**204**	**208**	**212**	**220**	**230**	**236**	**248**
Leptotene	83.6	35.5	6.3	4.9									
Zygotene	16.4	42.7	14.8	7.2									
Pachytene		14.2	31.3	13.6	9.6	4.0							
Diplotene		5.5	24.4	32.3	22.0	9.2							
Diakinesis		2.1	15.2	25.4	32.4	19.3	3.6	2.3					
Metaphase I			8.0	13.2	21.5	25.7	7.5	7.7	4.0				
Anaphase I				3.4	12.1	21.9	30.2	11.2	7.5	5.9			
Telophase I					2.4	9.6	26.8	26.3	17.6	9.3	4.5		
Prophase II						6.2	16.5	23.9	26.8	16.9	7.8	3.9	
Metaphase II						4.1	9.9	15.7	23.7	39.6	22.3	14.0	
Anaphase II							4.7	9.6	12.6	13.1	29.3	21.7	3.5
Telophase II							0.8	3.3	5.2	11.8	23.5	35.6	6.8
Tetrad									2.6	3.4	12.6	24.8	89.7

Meiosis of the PMCs in the triploid hybrid clone ‘Beilinxiongzhu 1#’ started 142 h after culture in the greenhouse. About 84% of the PMCs had fine filamentous chromosomes, which were at leptotene (Fig. 1a). The dominant meiotic stage changed from zygotene to diplotene at 168–188 h of culture (Fig. 1b–d). Monovalents, divalents, and trivalents were observed at diakinesis (Fig. 1e), indicating complex chromosome pairing in this triploid hybrid clone. Metaphase I became the dominant stage when male floral branches were cultured for 196 h (25.7%). Chromosomes of most PMCs were arranged on the equatorial plate at metaphase I, while a few PMCs had chromosomes free from the equatorial plate (Fig. 1f), which led to the unequal separation of chromosomes during anaphase I (30.2%; Fig. 1g–h), resulting in lagging chromosomes (Fig. 1g) and a chromosome bridge (Fig. 1h). After 208 h of culture, telophase I (Fig. 1i) was detected under light microscopy, indicating that the first cell division was complete. PMCs with a micronucleus (Fig. 1j) were observed during telophase I. In total, about 66 h were required for the PMCs to complete the first division.

Most of the PMCs directly initiated the second division without cytokinesis. Prophase II (Fig. 1k) became the dominant stage after 212 h of culture (26.80%). Some other meiotic stages were also observed, such as metaphase I, anaphase I, telophase I, and metaphase II, as shown in Fig. 1l–n, anaphase II as shown in Fig. 1o, telophase II as shown in Fig. 1p, and tetrads as shown in Fig. 1q. The PMCs contained parallel (Fig. 1m) fused spindles at metaphase II (Fig. 1n) besides the normally oriented spindles. Lagging chromosomes appeared during anaphase II (Fig. 1o). The dominant meiotic stage was telophase II (35.6%, Fig. 1p) and 24.8% of the PMCs developed into tetrads after 236 h of culture (Fig. 1q). Dyads (Fig. 1r), triads (Fig. 1s), and polyads (Fig. 1t) were observed at the tetrad stage. The second division was much shorter than the first division, taking only 36 h in total.

### Cytokinesis analysis of the PMCs in the triploid hybrid clone ‘Beilinxiongzhu 1#’

**Fig. 2:**
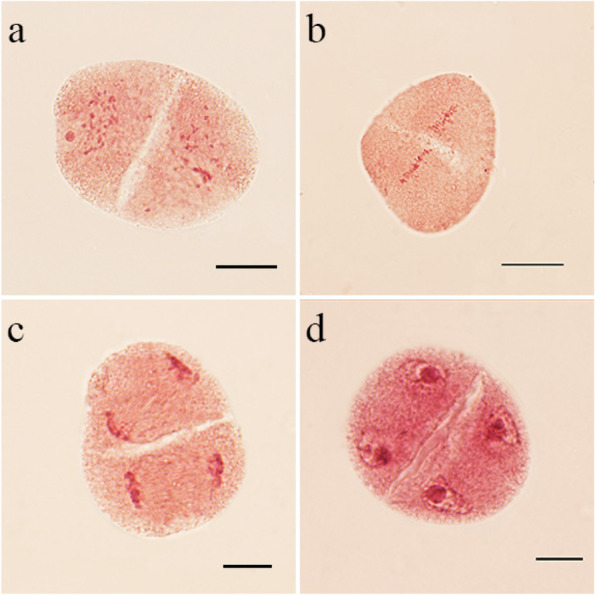
Premature cytokinesis during the second meiotic division of the PMCs in the triploid hybrid ‘Beilinxiongzhu 1#’. **a** PMCs undergoing premature cytokinesis at prophase II. **b** PMCs undergoing premature cytokinesis at metaphase II. **c** PMCs undergoing premature cytokinesis at anaphase II. **d** PMCs undergoing premature cytokinesis at telophase II. Bars, 10 μm

Meiotic cytokinesis of the PMCs has been divided into successive and simultaneous types [25]. Meiotic cytokinesis of the PMCs in *Populus* is the simultaneous type, which only occurs at the end of the second division. The cell plate did not form in the cytoplasmic domain during the second meiotic division (Fig. 1k–p). However, some triploid microsporocytes underwent premature cytokinesis following the first meiotic division as a result of the cell plate forming to divide the two daughter nuclei into distinct cytoplasmic domains (Fig. 2a). Chromosomes of the different domains are located in the equatorial region during the second meiosis, and separated into two different poles to form daughter nuclei (Fig. 2b–d). Premature cytokinesis was observed at different meiotic stages in the triploid PMCs (Table 2). The percentages of PMCs with premature cytokinesis in prophase II, metaphase II (Fig. 2b), anaphase II (Fig. 2c), and telophase II (Fig. 2d) were 45.0%, 62.0%, 68.3%, and 69.1%, respectively. This increased percentage of PMCs that were undergoing premature cytokinesis indicates that premature cytokinesis also occurred during the second meiotic division.

**Table 2:** Abnormalities in PMC cytokinesis in the triploid hybrid ‘Beilinxiongzhu 1#’

Meiotic stage of PMCs	Percentage of PMCs with premature cytokinesis /%
Prophase II	45.0 ± 5.0
Metaphase II	62.0 ± 12.2
Anaphase II	68.3 ± 4.9
Telophase II	69.1 ± 1.2

### Variations in pollen viability and diameter

**Fig. 3:**
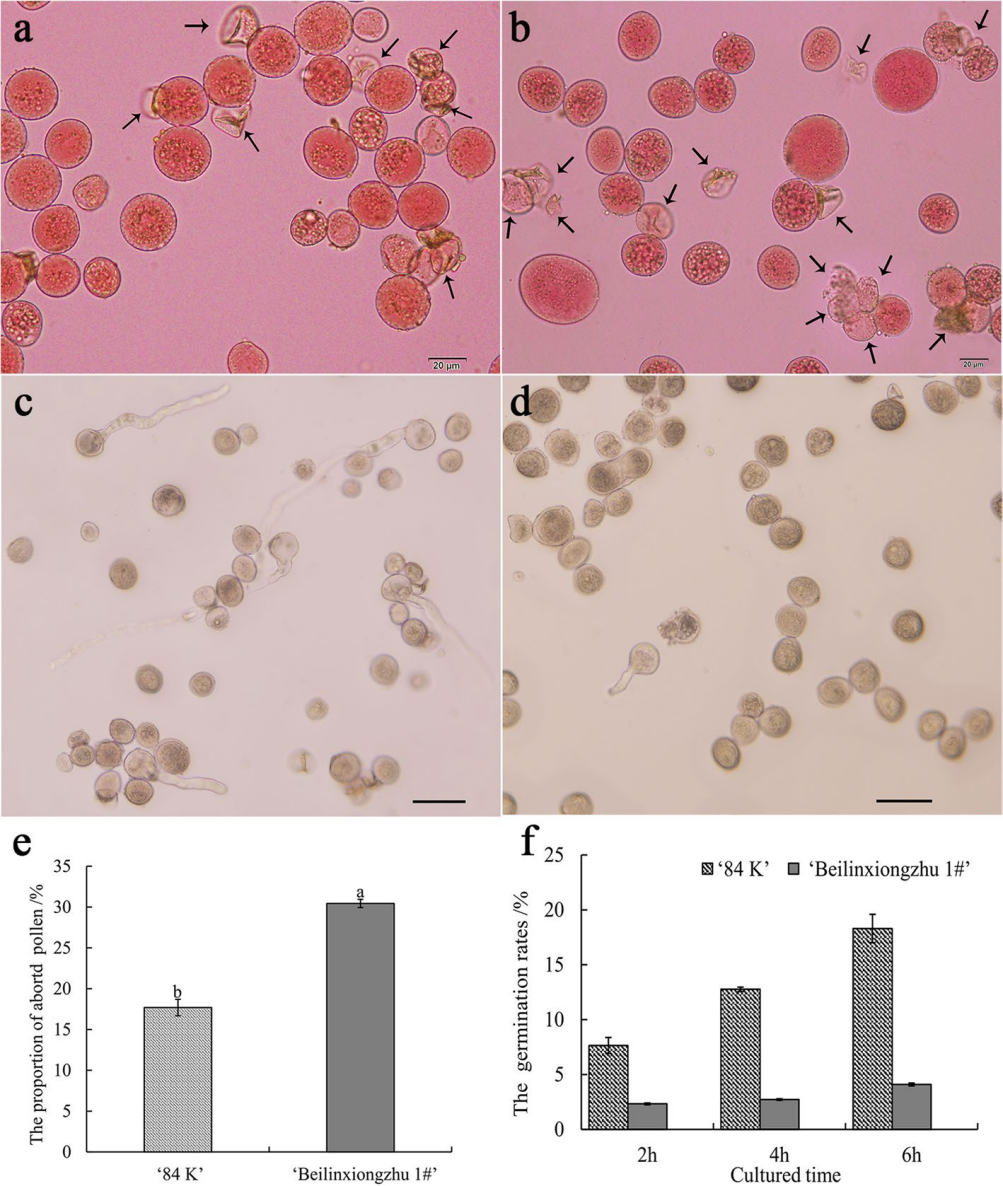
Variations in the pollen between the diploid hybrid ‘84 K’ and the triploid hybrid ‘Beilinxiongzhu 1#’. **a** ‘84 K’ pollen. Bars, 20 μm**. b** ‘Beilinxiongzhu 1#’ pollen. Bars, 20 μm. **c** Germinated ‘84 K’ pollen grains after 6 h of culture. Bars, 50 μm. **d** Germinated ‘Beilinxiongzhu 1#’ pollen grains after 6 h of culture. Bars, 50 μm. **e** The proportion of aborted pollen. **f** The pollen germination rates. Arrow indicates aborted pollen

Abnormal meiotic chromosomal behaviors are responsible for pollen viability in PMCs [26]. The pollen grains were stained with 2% aceto-carmine solution and the deeply stained pollen grains were regarded as fertile pollen. Unstained pollen grains were considered aborted (Fig. 3a–b). The percentages of aborted pollen grains by the diploid hybrid ‘84 K’ and the triploid hybrid clone ‘Beilinxiongzhu 1#’ are presented in Fig. 3e. The percentage of aborted pollen grains in the triploid hybrid clone ‘Beilinxiongzhu 1#’ was 30.44 ± 0.88%, which was significantly higher (*P* < 0.05) than that of diploid hybrid ‘84 K’. Furthermore, in vitro pollen germination experiments were carried out to evaluate pollen viability. As shown in Fig. 3f, the pollen germination rate of diploid hybrid ‘84 K’ increased gradually over time, and the highest pollen germination rate reached 18.30 ± 1.29% after 6 h of culture (Fig. 3c). However, a few germinated pollen grains were observed in the triploid hybrid clone ‘Beilinxiongzhu 1#’, and the pollen germination rate was 4.09 ± 0.22% after 6 h of culture (Fig. 3d), suggesting that pollen viability of the triploid hybrid clone ‘Beilinxiongzhu 1#’ was much lower than that of diploid hybrid ‘84 K’.

**Fig. 4:**
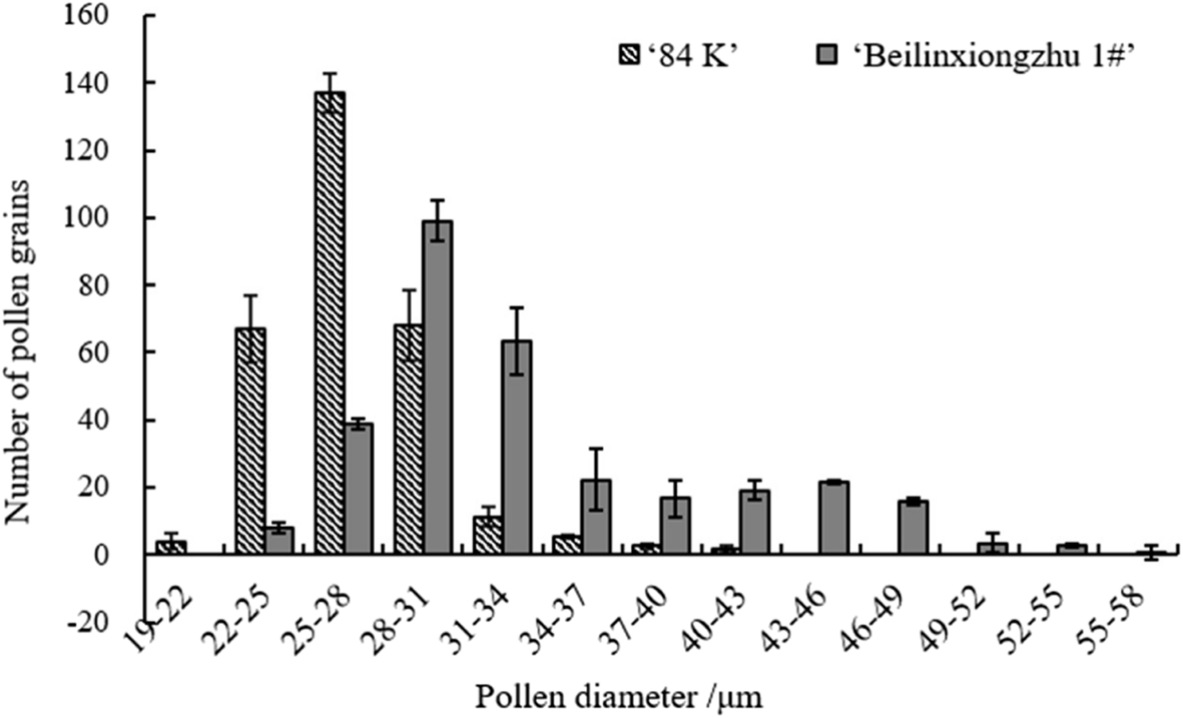
Histogram distribution of the pollen diameters of ‘84 K’ and ‘Beilinxiongzhu 1#’

The diameter of the pollen grains was closely correlated with the pollen genomic DNA content. The diameters of the pollen from the diploid hybrid ‘84 K’ and the triploid hybrid clone ‘Beilinxiongzhu 1#’ are presented in Fig. 4. The diameter of ‘84 K’ pollen ranged from 20.0 to 41.8 μm and the average diameter was 27.24 μm. The pollen diameters were normal distribution, and most pollen grains were 25–28 μm in diameter. The diameter of pollen in the triploid hybrid clone ‘Beilinxiongzhu 1#’ ranged from 22.5 to 55.0 μm, and the average diameter was 33.7 μm. Pollen diameters between 22 and 40 μm accounted for 85.85% of the total. In addition, some enlarged pollen grains (> 50 μm) were observed in the triploid hybrid clone ‘Beilinxiongzhu 1#’, suggesting that they may have higher ploidy levels than haploid pollen [27]. As shown in Fig. 4, the distribution of pollen diameter in the triploid hybrid clone ‘Beilinxiongzhu 1#’ was bimodal in the 37–40 μm range.

### Aneuploid progeny generated by crossing pollen from the triploid hybrid clone ‘Beilinxiongzhu 1#’

Although triploid plants are generally sterile due to their unbalanced chromosome segregation, a few pollen grains were fertile. To explore these fertile pollen grains in the triploid hybrid clone ‘Beilinxiongzhu 1#’, we crossed the pollen with female gametes from diploid hybrid ‘YXY 7#’ to produce a group of aneuploid progeny. A total of 4,510 seeds were obtained and 1,151 seeds germinated. The seed germination rate was 25.52%. A total of 393 seeds were sown and developed into young seedlings. Based on a pollen viability test and hybridization experiments, we concluded that triploid plants produced a small number of fertile gametes following complex meiosis.

### Detection of primary trisomy in the aneuploid progeny

Nineteen pairs of distinct SSR markers covering all 19 chromosomes screened from the 184 pairs of SSR markers were used to determine the number of chromosomes in aneuploid individuals. All polymorphic SSR markers were BLAST searched against the publicly available genome sequence of *P. trichocarpa* v4.1 (https://phytozome-next.jgi.doe.gov/info/Ptrichocarpa_v4_1) to determine the physical positions of the SSR loci. All 19 SSR primer pairs with two different alleles in the female parent and three distinct alleles in the male parent were screened out for genotyping of the aneuploid progeny. Detailed information on the 19 SSR primers is listed in Table 3. The SSR sequence, the chromosome number, the melting temperature (Tm), the motif sequences, and the physical position of each SSR pair in the parents are described.

**Table 3:** Information on the 19 pairs of SSR primers applied to detect the chromosomal composition of the backcross progeny

Name	Chromosome	SSR primer sequence	Tm/℃	Motif	Allele in female parent	Allele in male parent
PTSSR2102	1	Forward: CAGGGCATCACAGGAACAGA	59.7	[AG]_8_	233.1/237.1	233.1/235.0/237.1
		Reverse: TTAGTTGACACGCGTTGCAC	59.7			
Ptr-2-SSR56	2	Forward: TTGAAACAAGCTCAATCCTT	52.6	[AAG]_6_	356.8/365.1	356.8/365.1/368.0
		Reverse: AATGGTAATTTCCTGCTCAA	52.6			
GCPM-2151	3	Forward: TTCGTCATCGTTAATTTCAA	51.8	[AT]_14_	153.3/163.5	153.3/155.5/161.4
		Reverse: GTTGATTCATTGGGAAAATG	52.8			
MB71513	4	Forward: CCCGACAAGACAAATGTCAA	59.5	[AGA]_6_	254.2/257.5	254.2/257.3/260.4
		Reverse: CAAAACGGGTTGTTTTTGCT	60.0			
LG-V-2	5	Forward: AAAGAAACCAGACCACACAC	51.8	[TC]_13_	185.5/205.8	185.5/195.6/205.8
		Reverse: CGCTTGCCTTAATTAACAGT	53.1			
12	6	Forward: GGCTGCAGTCCCTGACAAA	58.9	[ATTTTT]_3_	248.0/298.5	235.6/237.2/243.4
		Reverse: AGATCTCGTGCATCGTGCA	57.6			
LG-III	7	Forward: GGATATGTCTCCACAAAGGA	51.9	[AC]_10_	274.0/278.2	274.0/278.2/284.0
		Reverse: GTACTGTCTCCGATACTGCC	51.8			
Ptr-8-SSR56	8	Forward: CAATGTTACTGCTGTCATGG	51.7	[TTG]_8_	366.3/377.1	347.9/377.3/379.4
		Reverse: TTATCTGCAAAGTTCACCCT	52.4			
PTSSR646	9	Forward: AATCCAGGTTGCACCGTGAA	60.2	[GCA]_6_	185.4/194.1	194.0/196.7/202.5
		Reverse: GTGCCACCGAAGAAGGAGAA	60.0			
Ptr-10-SSR12	10	Forward: CTTCGAGATCTGATGATGGT	51.7	[CGA]_6_	300.6/306.7	294.9/306.8/312.7
		Reverse: GTGAATCTCCGTTGTCAGTT	51.8			
SSR16	11	Forward: GTAGACAGCCTTTTGGTAAA	50.0	[AGG]_4_	195.7/201.6	195.8/198.6/201.5
		Reverse: GAATGAGACCCACTTTCACC	53.9			
Ptr-12-SSR53	12	Forward: AGCAAGAACTTACAGCAAGC	52.7	[TGC]_24_	232.2/244.9	226.0/232.2/244.9
		Reverse: CAACTTCTCCAATCTATGCC	52.2			
Ptr-13-SSR40	13	Forward: TGCTTATGGGTACAATGACA	51.9	[TC]_15_	277.7/294.2	277.7/284.5/296.1
		Reverse: GACCTCTGTAAACCCATTCA	52.1			
PTSSR1259	14	Forward: AAGGTTCGCTGGTAGGTTCG	60.0	[GGA]_5_	131.7/158.2	131.7/152.1/158.2
		Reverse: GCGGCAAAGAATCAGATCCG	59.7			
PTSSR1817	15	Forward: GGACTAAAAGGACCGGGTCG	60.1	[TGAT]_6_	250.0/257.4	257.5/261.5/269.5
		Reverse: CTCTCGCCTTCAGATCTCCG	59.7			
SSR8	16	Forward: GGTTAACCATAGCGAACGCAA	60.6	[CAAACA]_5_	238.3/241.0	233.4/238.3/246.4
		Reverse: GACGACCAACTCGGTGTCA	56.6			
MB70257	17	Forward: CGCGGGCTAATCAATAAGAT	59.2	[ATCT]_7_	279.0/283.1	275.2/279.0/294.3
		Reverse: TGAACAGAAACTTCGCTCCC	60.4			
GCPM-1577	18	Forward: GAGAACATGTCAGCAGTTCA	50.7	[TA]_11_	233.4/238.8	233.4/234.6/236.6
		Reverse: GCTTAAACATTGAGAAAGCG	53.4			
PTSSR1526	19	Forward: TTCCTCCGTGGTGGTTGATG	60.0	[GA]_9_	262.6/264.2	255.1/264.2/266.0
		Reverse: CCTCCCCTCCTTTTAAGCCC	59.7			

**Fig. 5:**
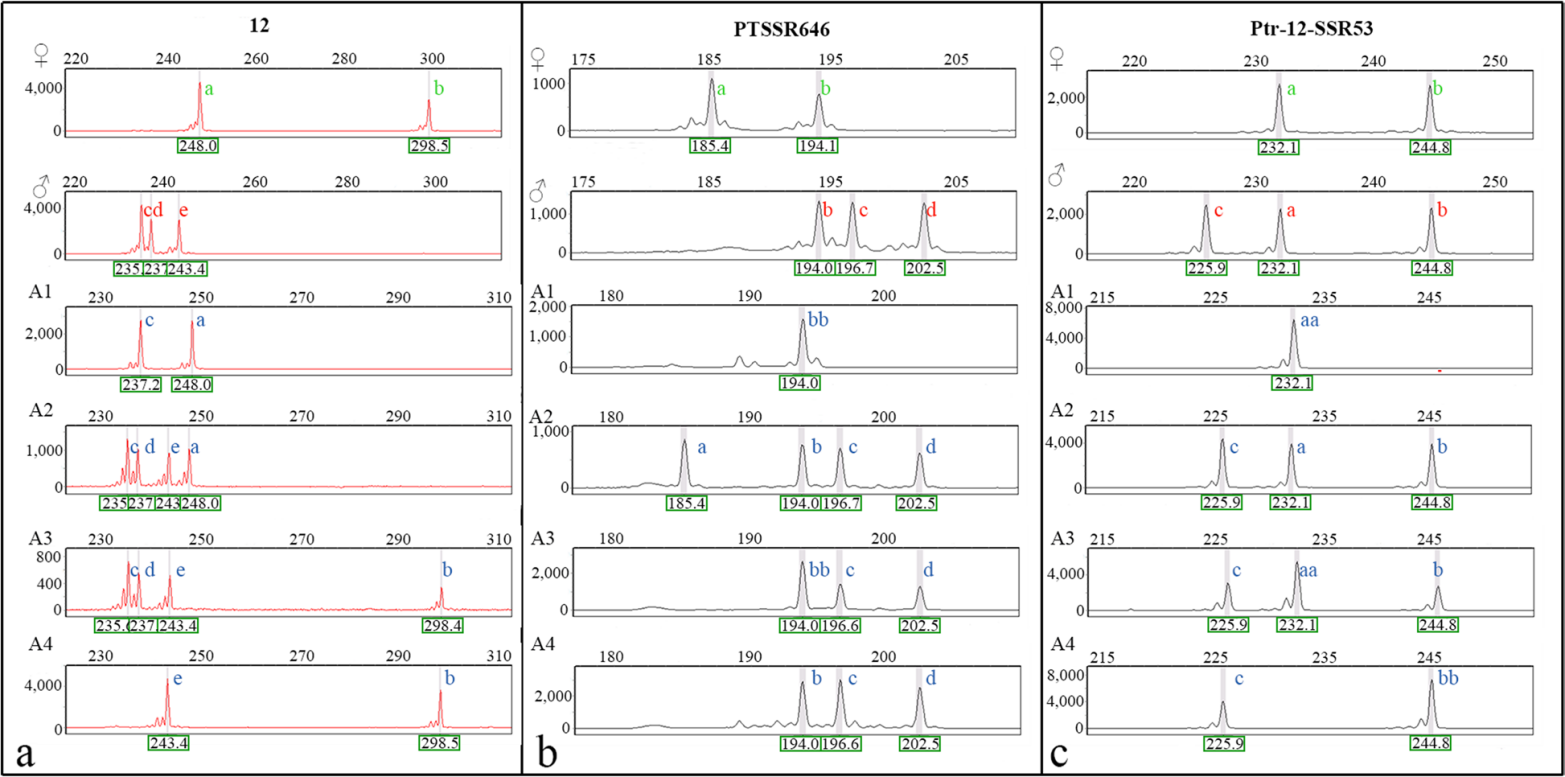
Capillary electrophoresis was used for the SSR markers to infer the chromosomal composition of the backcross progeny. **a** Genotypic analyses of the parents and some of the progeny based on capillary electrophoresis of locus 12. **b** Genotypic analyses of the parents and some of the progeny based on capillary electrophoresis of locus PTSSR646. **c** Genotypic analyses of the parents and some of the progeny based on capillary electrophoresis of locus Ptr-12-SSR53. Letters a–e represent the alleles at those loci. Green letters indicate the allelic composition of the female parent, red letters indicate the allelic composition of the male parent, and blue indicates the allelic composition of the progeny

Based on the number of shared alleles between the female parent ‘YXY 7#’ and the male parent ‘Beilinxiongzhu 1#’, all 19 SSR primer pairs were divided into three types. In the first type, both parents were heterozygous and had distinct alleles. Using primer 12 as an example, the parents ‘YXY 7#’ and ‘Beilinxiongzhu 1#’ were heterozygous with ‘ab’ and ‘cde’ genotypes, respectively (Fig. 5a). The number of distinct alleles (size in bp) at the 12 loci was equal to the number of chromosome 6 for each progeny. The allelic configurations found in aneuploids A1, A2, A3, and A4 were “ac”, “acde”, “bcde”, and “be”. Therefore, we determined that A1 and A4 had 2 chromosome 6 s, whereas A2 and A3 had 4 chromosome 6 s, respectively. In the second type, both parents were heterozygous and shared one distinct allele PTSSR646 is an example of this type (Fig. 5b). The parents ‘YXY 7#’ and ‘Beilinxiongzhu 1#’ were heterozygous, with ‘ab’ and ‘bcd’ genotypes, and they shared one distinct allele at the PTSSR646 locus (194.0 bp). The allelic configurations of aneuploids A1, A2, A3, and A4 were “bb”, “abcd”, “bbcd”, and “bcd”. These results show that the number of chromosome 9 s was 2, 4, 4, and 3 for A1, A2, A3, and A4, respectively. In the third type, both parents were heterozygous and shared two distinct alleles (Fig. 5c). For example, at the Ptr-12-SSR53 locus, the female parent ‘YXY 7#’ had heterozygous genotype ‘ab’, and the male parent ‘Beilinxiongzhu 1#’ was heterozygous with the genotype ‘abc’. The allelic configurations of aneuploids A1, A2, A3, and A4 were “aa”, “abc”, “aabc”, and “bbc”. Thus, we inferred that the number of chromosome 12 s was 2, 3, 4, and 3 for A1, A2, A3, and A4, respectively.

**Fig. 6:**
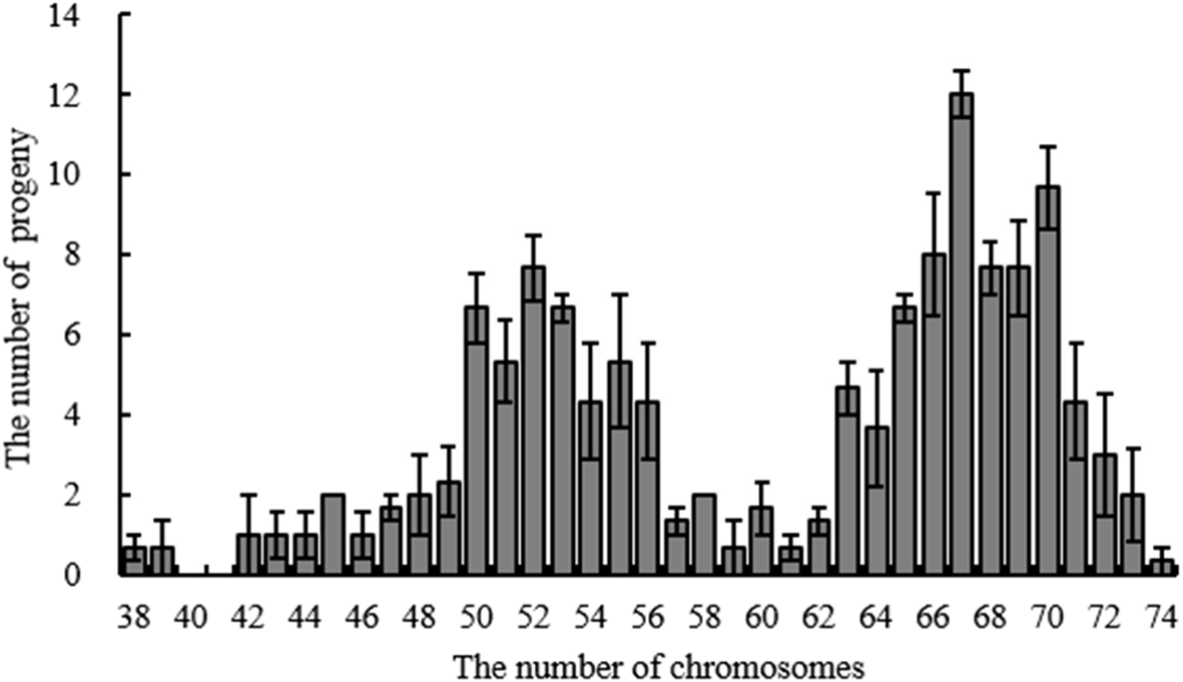
Histogram distribution of the number of chromosomes in the backcross progeny

Among all 393 progeny, the female parent ‘YXY 7#’ only contributed one allelic locus, and the other allelic locus was derived from the male parent ‘Beilinxiongzhu 1#’. According to the relative allelic dosage estimate from MAC-PR analysis at all loci for each sample, the number of chromosomes in the 393 backcross progeny determined by the 19 pairs of SSR primers covered the whole *Populus* genome. As shown in Fig. 6, the number of chromosomes of each aneuploid progeny ranged from 38 to 74. Similar to the diameter of the pollen grains, the total chromosome number of aneuploid progeny was bimodally distributed with a trough at 59. The chromosome number of most aneuploids varied between 63 and 71. Based on the estimated number of chromosomes, two diploids, 152 hyper-diploids, 23 hypo-triploids, one hyper-triploid, and 215 hypo-tetraploids were found.

**Fig. 7:**
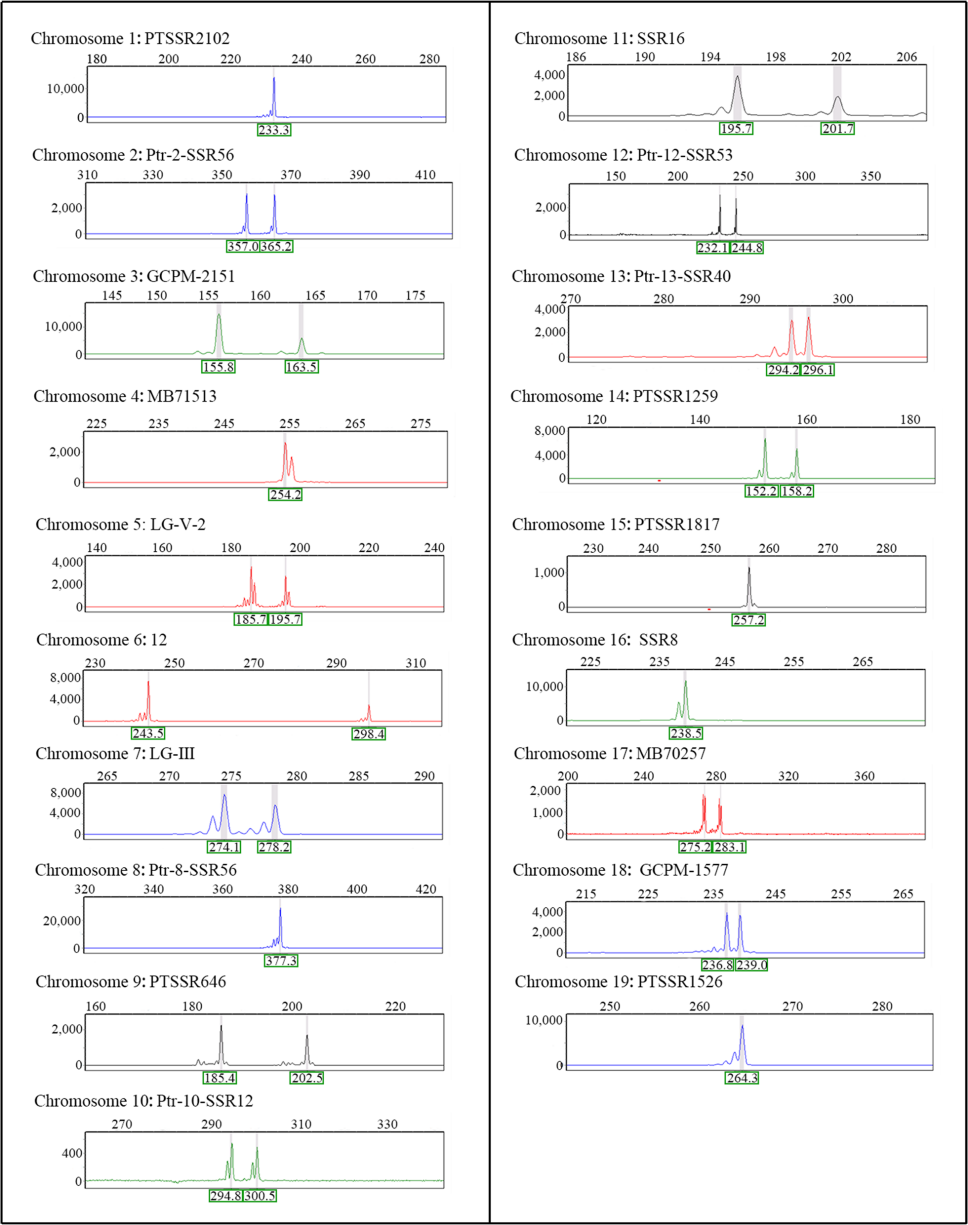
Capillary electrophoresis results of trisomy derived from chromosome 11

**Fig. 8:**
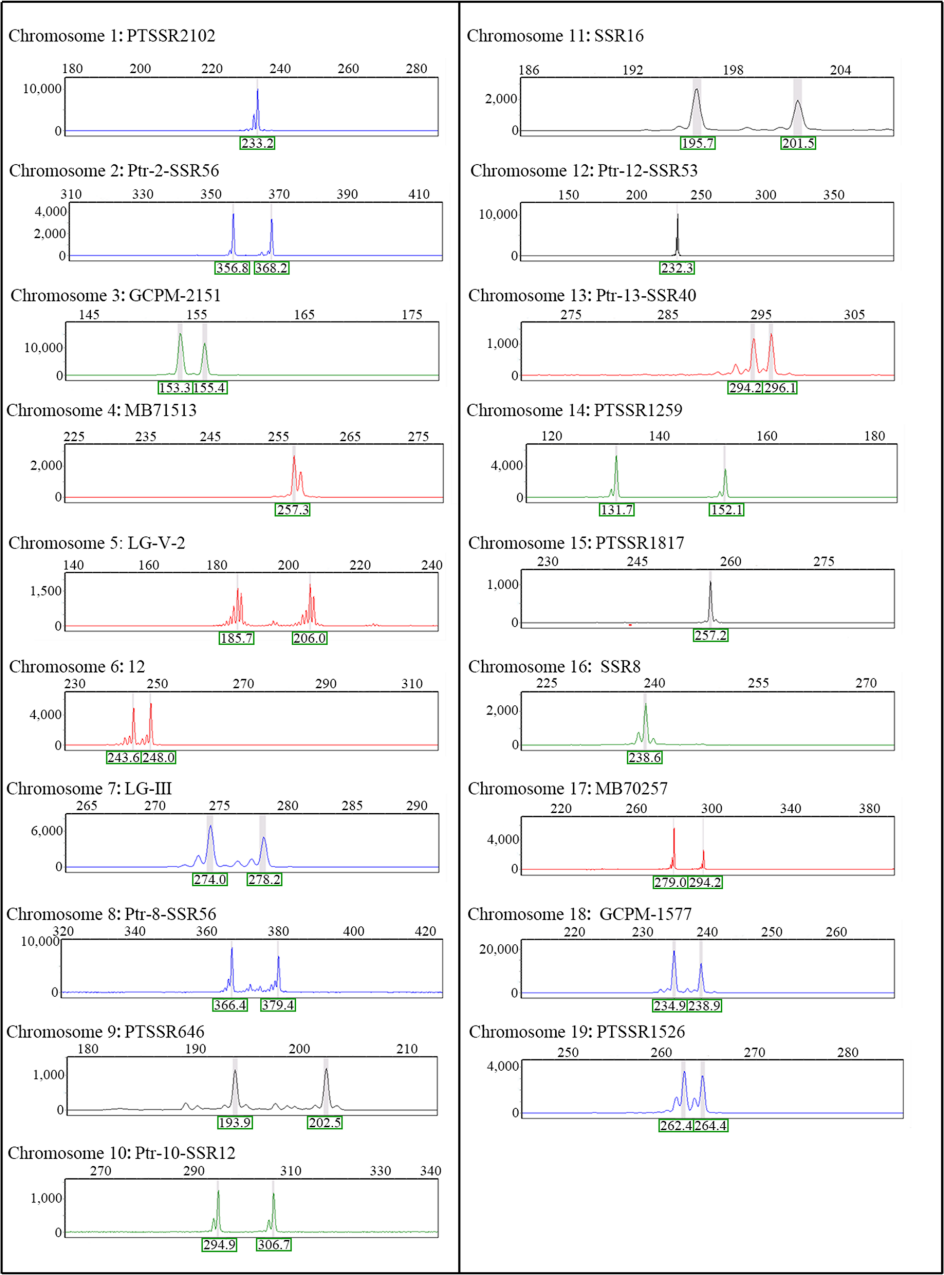
Capillary electrophoresis results of trisomy derived from chromosome 17

**Fig. 9:**
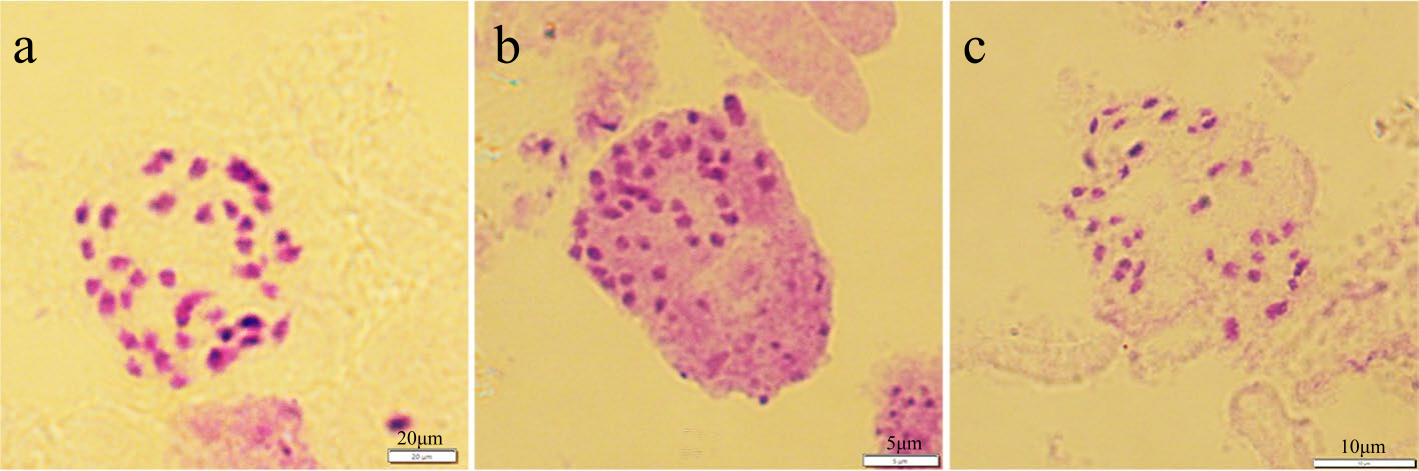
Somatic chromosome counts of aneuploid progeny and the diploid control. **a** Chromosome number of diploid ‘84 K’ (2n = 2*x* = 38). Bars, 20 μm. **b** Chromosome number of trisomy 11 (2n + 1 = 39). Bars, 5 μm. **c** Chromosome number of trisomy 17 (2n + 1 = 39). Bars, 10 μm

Among the 152 hyper-diploids, two putative primary trisomies derived from chromosome 11 (Fig. 7) and chromosome 17 (Fig. 8) were revealed by the allelic configurations of the 19 primer pairs. Trisomy 11 had three allelic loci on chromosome 11. Similarly, trisomy 17 had three allelic loci on chromosome 17. Subsequently, the two putative trisomies were confirmed by counting the number of somatic chromosomes. The chromosome number in the diploids was 38 (2n = 2*x* = 38, Fig. 9a), and the number of chromosomes in the trisomies was 39 (2n + 1 = 39, Fig. 9b–c), indicating that the two putative primary trisomies were real trisomies.

## Discussion

Triploids normally contain three copies of each chromosome. Therefore, complex chromosome pairing and chromosome segregation occur during meiosis. In this study, high frequencies of meiotic abnormalities were detected in triploid poplar, including univalents, premature migration of chromosomes, lagging chromosomes, chromosome bridges, and micronuclei, indicating a high genetic imbalance in this triploid. Microspores with aneuploid chromosome sets formed as a result of unbalanced chromosome segregation and elimination of chromosomes. The results were consistent with studies on other triploid poplar species, such as (*Populus tomentosa* × *P. bolleana*) × *P. tomentosa* [28] and (*P. tomentosa* × *P. bolleana*) × (*P. alba* × *P. glandulosa*) [27]. Furthermore, this meiotic behavior is similar to other triploid species, such as *Glycine max* L. [29], *Cucumis sativus* L. [30], and *Lilium pumilum* DC [26].

Studies have shown that cytokinesis of PMCs in *Populus* are of the simultaneous cytokinesis type [31–33]. Premature cytokinesis often occurs in *Populus tomentosa* and other triploid hybrids [27, 34, 35]. In the present study, premature cytokinesis was observed in the triploid PMCs during the second meiotic division (Fig. 2), and the frequency of PMCs with premature cytokinesis was significantly higher than that documented in previous reports. Therefore, we speculated that multiple factors may be partially responsible for premature cytokinesis.

Triploid cytogenetics studies have shown that low triploid fertility is associated with a high frequency of abnormal chromosomal behavior during meiosis [36–38]. In this study, the pollen of the triploid hybrid clone ‘Beilinxiongzhu 1#’ was fertile, and the pollen staining and germination rates were 69.5% and 2.9%. In addition, 4,510 seeds were obtained by pollinating ‘YXY 7#’ with ‘Beilinxiongzhu 1#’. A total of 1,151 seeds germinated and 393 seeds developed into young seedlings. These results indicate that lower fertility also occurred in the triploid hybrid ‘Beilinxiongzhu 1#’. Therefore, it was possible to generate primary trisomy with the 2*x* × 3 *x* hybrid.

Pollen grain size has been used as an indicator of pollen ploidy level, as cell size increases with DNA content [39]. The range of pollen size in triploids tends to be widely distributed, indicating that pollen grains with different chromosome numbers are produced due to irregular chromosome pairing and unbalanced separation of chromosomes [40, 41]. Most pollen grains produced by a hybrid triploid are aneuploid. The diameter of the pollen of the triploid hybrid clone ‘Beilinxiongzhu 1#’ varied significantly, ranging from 22.5 to 55.0 μm. In addition, the distribution of the pollen diameter was a bimodal curve with the through at 37–40 μm, which was similar to the chromosome distribution number of aneuploid progeny. However, the distribution of pollen diameter was clustered at the first peak compared with the distribution of the chromosome number.

In this study, we identified two primary trisomies using 19 pairs of SSR primers distributed on 19 chromosomes of *Populus* and somatic chromosome counted from 393 progeny. Although it is common for breeders to generate primary trisomy using a 2*x* × 3 *x* hybrid [9, 12, 13], this was the first time primary trisomy was obtained in a tree species. Compared with other methods to identify primary trisomy, this experimental method accurately identified the number of chromosomes [7, 20, 21], because this method required that all of the SSR markers were polymorphic between the hybrid parents and included all 19 *Populus* chromosomes. Additionally, the number of chromosomes for a large number of samples can be determined quickly using our method. However, a drawback of this approach is a long time needed to screen suitable polymorphic SSR markers.

## Conclusion

Many meiotic abnormalities, including premature migration of chromosomes, lagging chromosomes, chromosome bridges, asymmetrical separation, micronuclei, and premature cytokinesis were discovered during meiosis of the triploid hybrid clone ‘Beilinxiongzhu 1#’. However, these abnormal behaviors did not result in completely aborted pollen. The pollen diameter of the triploid hybrid clone ‘Beilinxiongzhu 1#’ was bimodally distributed, which was similar to the number of chromosomes. We provide a protocol for determining the number of chromosomes in aneuploid progeny and 19 distinct SSR primer pairs, including the entire *Populus* genome, were developed. Two primary trisomies were detected from 2*x* × 3 *x* hybrids using the SSR molecular markers and counting of the somatic chromosomes. Our findings provide a powerful genetic tool to reveal the function of *Populus* genes.

## Materials and methods

### Plant materials

The collection of flower branches were carried out in late January every year. Male floral branches of ‘Beilinxiongzhu 1#’ were collected from the Huayang Forest Tree Nursery, Hebei Province, China. Female branches of *P. alba* × *P. glandulosa* ‘YXY 7#’ (2n = 2*x* = 38) were collected from the Guan Country Nursery, Shandong Province. All branches were trimmed and cultured in tap water in a greenhouse (10–20 °C) at the Beijing Forestry University to force flower development. No extra nutrients were supplied to the tap water. Male buds of *P. alba* × *P. glandulosa* ‘84 K’ were used as the control group. Remarkably, we have permission to collect plant material used in the study. Kang Xiangyang, professor of National Engineering Research Center of Tree breeding and Ecological Restoration, undertook the formal identification of the plant material used in our paper. At present, all materials has been deposited in the Huayang Forest Tree Nursery, Hebei Province, China.

### Meiotic and cytokinesis analyses of the PMCs

The flower branches of ‘Beilinxiongzhu 1#’ and ‘84 K’ were cultured in a greenhouse. Two to three flower buds were randomly sampled every 2 h and fixed in Carnoy’s fluid (ethanol: acetic acid, 3:1) until tetrads appeared. After 24 h, the fixed buds were transferred in 70% ethanol solution and stored at 4 °C. PMCs were dissected from the anthers of fixed flower buds using forceps and were crushed in a droplet of acetocarmine solution (2%). Examine developing microsporocytes under fluorescence microscope with 10 × eyepiece and 100 × objective lens (model BX61; Olympus, Tokyo, Japan). And a CCD camera (model DP70; Olympus) were used to take pictures. This meiotic analysis of the PMCs was conducted following the method of Kang et al. [42]. Approximately 200–300 PMCs were counted in each sample to determine the dominant meiotic stages. A meiotic analytical method was used to observe cytokinesis of the PMCs.

### Measurement of pollen grain diameter

When the catkins of ‘Beilinxiongzhu 1#’ and ‘84 K’ matured, pollen samples were collected and stored in centrifuge tubes with allochronic silica gel at –20℃. The ‘Beilinxiongzhu 1#’ and ‘84 K’ pollen grains were stained with acetocarmine solution to prepare temporary smears. The pollen grains that were dyed red with the acetic acid magenta were viable, while the empty and unstained pollen grains were unviable [43]. The images of the pollen grains were taken using a CCD camera. In total, 300 viable pollen grains were randomly selected to determine the pollen diameter using ImageJ software (Version 1.53c). The experiment was repeated three times.

### Evaluation of pollen viability

Pollen was germinated in vitro to assess viability. The in vitro pollen germination medium of *Populus tomentosa* was prepared with reference to [44]. Fresh pollen grains of ‘Beilinxiongzhu 1#’ and ‘84 K’ were sampled and spread on slides containing the medium. The slides were placed in a Petri dish containing moist filter paper and cultured in a light incubator at 26℃, with an illumination level of 4 (1500 lx) and humidity of 40%. After 2, 4, and 6 h of culture, the pollen grains were fixed in Carnoy’s fluid (ethanol: acetic acid, 3:1), respectively. The germination rate of all samples was calculated using an eyepiece micrometer. The test was repeated three times, and at least 50 pollen grains were counted for each repetition.

### Aneuploid production by crossing ‘YXY 7#’ with ‘Beilinxiongzhu 1#’

When the stigmas of the ‘YXY 7#’ female flower buds were receptive, they were pollinated with fresh pollen from ‘Beilinxiongzhu 1#’. The female floral branches were further hydroponically cultured. The seeds matured after approximately 4 weeks of cultivation. They were harvested and sown in 54 × 28 × 10 cm nutrition plates in the greenhouse.

### Detection of primary trisomy

The genomic DNA of all samples was extracted from young leaves using a DP320 DNA Secure Plant Kit (Tian Gen, Beijing, China), following the manufacturer’s instructions. A photometer was used to assess the purity and concentration of the stock DNA. The quality and concentration of the stock DNA were checked with the NanoDrop 2000 spectrophotometer (Thermo Fisher Scientific, Wilmington, DE, USA). The SSR markers used for this study were polymorphic between the parents and included all chromosomes. According to this principle, SSR primers were selected from (1) the SSR database (https://web.ornl.gov/sci/ipgc/ssr_resources.htm) (beginning with ‘GCPM’) released by the International *Populus* Genome Consortium; (2) SSRs developed previously from an mRNA sequence in our lab (beginning with ‘PTSSR’ and ‘MB’), and (3) SSR primers developed from the *P. trichocarpa* genomic sequence (beginning with ‘LG’) [45]. The screened primers were applied to the number of aneuploid progeny.

The fluorescently labeled TP-M13-SSR method [46] was employed. The 5′-forward primer was attached with a universal primer M13 tail (5′-TGTAAAACGACGGCCAGT-3′) and labeled with four fluorescent substances (6-carboxy-X-rhodamine, 6-carboxy-fluorescein, tetramethyl-6-carboxyrhodamine, or 5-hexachloro-fluorescein). All primers were synthesized by Sangon Biotech Co. Ltd. (Shanghai, China). The PCR amplification protocol was 5 min at 94℃, 10 cycles of 30 s at 94℃, 30 s at the optimal annealing temperature for each SSR marker, and 30 s at 72℃; 25 cycles of 30 s at 94℃, 30 s at 53℃, and 30 s at 72℃, with a final extension of 8 min at 72℃. The PCR products were used for the capillary electrophoresis fluorescence-based SSR analysis with the ABI 3730XL DNA Analyzer (Applied Biosystems, Foster City, CA, USA), and fragment sizes and peak areas were analyzed using GeneMarker 1.75 software [47] to determine the number of chromosomes in each individual.

### Somatic chromosome counts

The number of chromosomes in each plantlet was ultimately confirmed by counting the number of somatic chromosomes. The stem tips were cut from the seedlings and pretreated in a saturated solution of paradichlorobenzene for 3 h. The pretreated samples were washed once and fixed in Carnoy’s fluid (ethanol: acetic acid, 3:1) for at least 24 h at 4℃. The fixed stem tips were hydrolyzed for 25 min at room temperature in 38% HCl, followed by three rinses in distilled water for 10 min. The hydrolyzed samples were stained with Carbol fuchsin, crushed with a coverslip, and examined under an Olympus BX61 microscope at 100 × under an oil lens.

### Statistical analysis

Analysis of variance was conducted using the UNIVARIATE procedure in SPSS software (SPSS for Windows, version 20; SPSS Inc., Chicago, IL, USA). Excel software (version 2206 Build 16.0.15330.20260, Microsoft Inc., Redmond, WA, USA) was used to assist with the statistical analysis and drawing. A *P*-value < 0.05 was considered significant.

## Data Availability

All data generated or analyzed during this study are included in this published article.
